# Early-Life Galacto-Oligosaccharide Supplementation Induces Persistent Immunoglobulin and Metabolic Alterations in Holstein Dairy Calves by Shaping Gut Microbiota

**DOI:** 10.3390/ani16010126

**Published:** 2026-01-01

**Authors:** Qi Huang, Meinan Chang, Peng Sun

**Affiliations:** State Key Laboratory of Animal Nutrition and Feeding, Institute of Animal Science, Chinese Academy of Agricultural Sciences, Beijing 100193, China; workhq@163.com (Q.H.); meinan0616@126.com (M.C.)

**Keywords:** galacto-oligosaccharides, immunity, gut microbiota, dairy calves, metabolomics

## Abstract

Early-life development of immune functions is crucial for calf health, growth, and future productivity. Galacto-oligosaccharides (GOSs) have been reported to facilitate ruminal microbial establishment and improve growth in Holstein dairy calves, but their prolonged influence on immunoglobulin levels, hindgut microbiota, and metabolic regulation remains insufficiently understood. This study evaluated the effects of early-life GOS supplementation on immune-related indicators, intestinal microbial ecology, and metabolic profiles in Holstein calves. Twenty-four newborn Holstein female dairy calves were randomly assigned to a control group (CON, *n* = 12) or a GOS group (GOS, *n* = 12; 10 g/day from birth to day 28). After supplementation ceased on day 28, calves previously receiving GOS were referred to as the GOSS group (*n* = 6). Immunoglobulin levels, gut microbiota, and fecal and serum metabolomes were evaluated during supplementation and six weeks after withdrawal. GOS supplementation significantly increased serum IgA and IgG levels during the treatment, with IgG levels remaining elevated for six weeks after discontinued supplementation. Although overall microbial diversity was not markedly altered, GOS selectively enriched bacterial taxa and function pathways linked to amino acid synthesis, unsaturated fatty acid production, and coenzyme-related metabolism. On day 70, GOSS group displayed distinct fecal and serum metabolomic profiles, with altered metabolites primarily associated with vitamin B6, folate, cobalamin metabolism, branched-chain amino acid biosynthesis, and purine and arginine pathways. These results demonstrate that early-life GOS supplementation promotes sustained immune and metabolic alterations following supplementation cessation, potentially mediated by modulation of gut microbial functions. These findings suggest that early dietary GOS supplementation may support physiological maturation in calves and could be useful as a nutritional strategy in calf-rearing systems.

## Simple Summary

Dairy calves rely on a well-developed immune system and healthy gut to grow strong and stay productive later in life. We investigated whether supplementing galacto-oligosaccharides in the early diet of calves influences immunoglobulin levels, gut microbiota, and metabolism after supplementation stops. Calves that received this ingredient showed higher levels of immunoglobulin in their blood, and these benefits continued even after the supplement was no longer given. Although overall microbial diversity was not greatly altered, galacto-oligosaccharides promoted the enrichment of beneficial bacterial taxa and metabolic pathways linked to amino acid synthesis, lipid metabolism, and cofactor production. By day 70, calves supplemented with galacto-oligosaccharides early in life displayed distinct fecal and serum metabolite profiles, particularly in pathways related to vitamin B6, folate and cobalamin metabolism, branched-chain amino acid biosynthesis, and purine and arginine metabolism. These findings demonstrate that early galacto-oligosaccharides supplementation can influence host immunity, gut microbial functions, and metabolic profiles. Such early nutritional strategies may help improve calf growth performance, reduce disease susceptibility, and support more sustainable dairy production.

## 1. Introduction

Early-life nutrition strategies are pivotal in shaping the long-term health, growth performance, and immune competence of dairy calves [1]. The neonatal stage is a particularly sensitive period, during which both immune system function and gut microbial communities are still developing and highly susceptible to dietary influences [2,3,4]. Establishment of the gut microbiota during this phase plays a vital role in promoting metabolic function maturation, the strengthening of immune defenses, and the ability to resist disease [5]. Thus, nutritional strategies that promote beneficial microbial establishment and immune function during this stage are essential for improving calf health, reducing disease susceptibility, and enhancing lifetime productivity [6].

Galacto-oligosaccharides (GOSs) are naturally occurring functional oligosaccharides found in milk and are recognized for their capacity to influence gut microbial ecology and support immune development in neonates [7]. Since GOS cannot be broken down in the upper digestive tract [8], they reach the hindgut largely unaltered and selectively promote the proliferation of beneficial microbes, including *Bifidobacterium* and *Lactobacillus* [9]. Acting as prebiotics, previous studies indicate that GOS enhance intestinal integrity, limit pathogenic colonization, modulate immune responses, and improve nutrient utilization [10,11]. These beneficial outcomes are thought to result primarily from enhanced short-chain fatty acid (SCFA) production, strengthened gut barrier function, and regulation of immune-related signaling pathways [12].

In dairy calves, emerging evidence supports the benefits of GOS during early life. For instance, Chang et al. [13] observed that GOS supplementation enhanced growth and reshaped rumen microbiota, while Yu et al. [14] demonstrated enhanced growth performance and immune function. However, whether these effects persist after GOS withdrawal and how early-life GOS supplementation influences hindgut microbial composition, microbial metabolic function, and host metabolic profiles remain unclear. In addition, the relationships linking GOS-associated microbial changes with immune-related and metabolic responses in ruminants are not fully understood.

Given the potential of early-life nutritional intervention to exert lasting effects, the present study aimed to evaluate the effects of early-life GOS supplementation on immune-related indicators, hindgut microbial ecology, and both fecal and serum metabolomic profiles in Holstein calves. These findings provide new insights into how early prebiotic intervention may promote immunoglobulin levels and host–microbiota metabolic interactions, with implications for post-supplementation effects of GOS and productivity in dairy systems.

## 2. Materials and Methods

### 2.1. Animal Study and Sample Collection

Twenty-four newborn Holstein female dairy calves with similar initial body weights (mean ± SEM: 37.8 ± 0.90 kg) were randomly assigned to one of two treatments: a control group (CON; *n* = 12), provided with a basal diet only, or a GOS group (*n* = 12), supplemented daily with 10 g GOS (purity ≥ 90%; Quantum Hi-Tech Biological Co., Guangzhou, China). This GOS dosage of 10 g/day was selected based on our previous work showing that 10 g/day effectively enhanced growth performance, reduced diarrhea incidence, and promoted rumen microbial colonization in preweaning Holstein calves [13]. After 28 days of treatment, calves in the GOS group were further divided into two subsets: one ceased supplementation (GOSS; *n* = 6) and the other maintained daily GOS intake (GOSC; *n* = 6). Immediately post-calving, all calves were separated from their dams and reared individually in pens (1.8 × 1.4 × 1.2 m) to avoid microbial cross-contact. Each calf received 4 L of pooled colostrum by bottle-feeding within 1 h after birth, ensuring uniform colostrum source and intake across all animals. On days 2 to 3, calves received colostrum in three daily feedings (6 L/day total; 06:00, 14:00, 18:00). From day 4 until day 70, animals were fed 8 L/day of farm-collected raw milk that was heat-treated at 60 °C and cooled to 38–39 °C prior to feeding. A commercial starter (Tianjin Jiuzhou Dadi Feed Co., Tianjin, China) was offered from day 3 onward. All calves received identical feedstuffs and management throughout the study; nutritional composition details are given in Table S1 according to Chang et al. [13]. Calves gradually weaned and reached weaning at 70 days of age. All calves were managed under identical health protocols (nutrition, vaccinations, anthelmintic treatments, etc.) and remained healthy without antibiotic treatments throughout the study.

Approximately 1.5 g of fresh feces was obtained directly from the rectum on days 28 and 70, immediately frozen in liquid nitrogen (−196 °C), and stored at −80 °C until analysis. Blood samples were collected from the jugular vein using vacuum blood collection tubes without anticoagulant (vacutainer; BD Biosciences, San Jose, CA, USA) before morning feeding on days 7, 14, 28, 42, 56, and 70, left to clot for 30 min at room temperature, and centrifuged (3000× *g* for 15 min, 4 °C) to obtain serum. The separated serum was stored at −80 °C until analysis.

### 2.2. Serum Immunoglobulin Assay

Concentrations of serum immunoglobulins IgA, IgG, and IgM were determined using commercial ELISA kits (Bethyl Laboratories, Montgomery, TX, USA; catalog numbers E11-131, E11-118, and E11-101, respectively). All procedures followed the instructions provided by the manufacturer [15].

### 2.3. Extraction of Fecal Microbial DNA

After thawing on ice, total bacterial DNA was extracted from 0.20 to 0.50 g of fecal material using a Mag-Bind Soil DNA Kit (Omega Bio-Tek, Norcross, GA, USA) according to the manufacturer’s protocol. DNA concentration and purity were assessed using a Qubit 3.0 fluorometer (Invitrogen, Carlsbad, CA, USA), and integrity was confirmed by electrophoresis on a 2% agarose gel.

### 2.4. 16S rRNA Gene Sequencing

The V3–V4 region of the bacterial 16S rRNA gene was amplified using primers 341F (5′-CCTACGGGNGGCWGCAG-3′) and 805R (5′-GACTACHVGGGTATCTAATCC-3′). The first-round PCR (30 μL total volume) contained 15 μL of 2× Hieff^®^ Robust PCR Master Mix (Yeasen Biotechnology, Shanghai, China), 1 μL of each primer, 10–20 ng template DNA, and nuclease-free water. The thermal program comprised 94 °C for 3 min, followed by two-step cycling: The first phase included five cycles with 94 °C (30 s), 45 °C (20 s), and 65 °C (30 s); then 20 cycles of 94 °C (20 s), 55 °C (20 s), and 72 °C (30 s); ending with a 5 min elongation step at 72 °C. The second PCR was conducted to attach Illumina sequencing adapters, using 20–30 ng of the first PCR product in a similar 30 μL reaction system. The amplification steps consisted of 95 °C for 3 min; five cycles of 94 °C (20 s), 55 °C (20 s), 72 °C (30 s); and a 5 min final extension at 72 °C. Amplicons were purified with a DNA Purification Kit (Axygen Biosciences, Union City, CA, USA) and sequencing libraries were prepared using the TruSeq DNA Library Prep Kit (Illumina, San Diego, CA, USA). Paired-end sequencing was conducted on an Illumina MiSeq platform (Illumina, San Diego, CA, USA).

### 2.5. Bioinformatics Analysis of Microbiome

Raw reads were assembled using PEAR (v0.9.8) and further analyzed within the QIIME2 platform [16]. After demultiplexing (demux plugin) and primer removal (cutadapt v1.18) [17], the high-quality sequences were denoised and chimera-filtered using the DADA2 pipeline (v1.14.0) [18] to generate representative amplicon sequence variants (ASVs). Multiple sequence alignment was carried out by MAFFT (v7.450) [19,20], and a phylogenetic tree was inferred with FastTree (v2.1.7) [21]. Alpha diversity indices were calculated using Mothur (v3.8.31) [22], whereas beta diversity patterns were evaluated with Bray–Curtis distance and visualized through PCoA using the vegan package in R (v2.5-6). Statistical comparisons among groups were performed using ANOSIM. Taxonomic differences were examined by Welch’s *t*-test and linear discriminant effect size (LEfSe) analysis [23], applying an LDA threshold of three. Functional potential was inferred using PICRUSt2 based on KEGG and MetaCyc reference pathways, and pathway-level differences were determined by Wilcoxon rank-sum tests in STAMP [24].

### 2.6. Metabolomics Analysis

Metabolomic profiling of fecal and serum samples was performed using UPLC/MS. Each sample (0.6 mL) was extracted with pre-cooled methanol containing 2-chlorophenylalanine (4 ppm) as an internal reference, then homogenized by vortexing and bead-beating. The mixtures were sonicated for 10 min and centrifuged at 12,000× *g* for 10 min at 4 °C. Supernatants (300 μL) were filtered through 0.22 μm membranes before injection. To monitor instrument drift, pooled quality control (QC) samples were generated by combining small aliquots of all extracts. Chromatographic separation was carried out on a Waters ACQUITY HSS T3 column (2.1 × 150 mm, 1.8 μm) using a Thermo U3000 UHPLC system (Thermo Fisher Scientific, Dreieich, Germany) coupled to a Q Exactive Plus mass spectrometer (Thermo Fisher Scientific, Dreieich, Germany).

Analyses were conducted under both positive and negative electrospray ionization modes under specified gradient conditions. The autosampler was maintained at 8 °C, with a flow rate of 0.25 mL/min, a column temperature of 40 °C, and a 2 μL injection volume. The mobile phase consisted of water and acetonitrile containing either 0.1% formic acid (for positive mode) or 5 mM ammonium formate (for negative mode). Gradient elution was programmed from 2% to 98% organic solvent over 12 min, followed by re-equilibration. The MS source parameters were set as follows: spray voltage at 3.5 kV (positive mode) or 2.5 kV (negative mode); sheath and auxiliary gases at 30 and 10 arb, respectively; capillary temperature at 325 °C. Full-scan spectra were acquired at 70,000 resolution within an *m*/*z* range of 81–1000. MS/MS data were collected using higher-energy collisional dissociation (HCD, 30 eV). Dynamic exclusion was enabled in order to avoid repeated ion acquisition.

### 2.7. Bioinformatics Analysis of Metabolomics

According to Rasmussen et al. [25], the LC–MS/MS raw files were converted to mzXML format through MSConvert (ProteoWizard v3.0.8789) and analyzed with the R package XCMS (v3.12.0) for peak extraction, alignment, and correction of retention time (key parameters: peakwidth = c (5, 30), method = “centWave”, mzwid = 0.015, ppm = 15, bw = 2, mzdiff = 0.01) [26]. Features exhibiting relative standard deviations (RSDs) above 30% in pooled QC samples were excluded before downstream analysis. Metabolite annotation was performed by comparison with several public databases, including HMDB [27], KEGG [28], MassBank [29], mzCloud [30], and LipidMaps [31], using accurate mass and MS/MS spectral similarity.

The principal component analysis (PCA) and orthogonal partial least squares-discriminant analysis (OPLS-DA) were performed using the ropls package (v1.22.0) in R [32]. Significantly altered metabolites were selected based on variable importance in projection (VIP > 1) values from the OPLS-DA model and Student’s *t*-test results (*p* < 0.05). Enrichment and topological pathway analyses were then implemented using hypergeometric testing, with significant compounds mapped to KEGG pathways to interpret metabolic perturbations [33].

### 2.8. Statistical Analysis

Data for immunoglobulins (IgA, IgG, IgM) were analyzed using Student’s *t*-test for pairwise comparisons or one-way ANOVA followed by Tukey’s multiple comparison test (SPSS 26.0; IBM, Corp., Armonk, NY, USA). Alpha diversity indices were compared using the Wilcoxon rank-sum test, whereas taxonomic differences were determined by Welch’s *t*-test and LEfSe. Functional pathway differences (KEGG and MetaCyc) were also analyzed using Wilcoxon tests. Statistical significance was defined as *p* < 0.05. Spearman’s rank correlation was used to assess associations across variables, with Spearman’s |R| > 0.50 and *p* < 0.05 considered significant. Co-expression networks were visualized and analyzed in Cytoscape (v3.8.0).

## 3. Results

### 3.1. Dietary GOS Supplementation Persistently Promotes Circulating Immunoglobulin Levels

**Figure 1:**
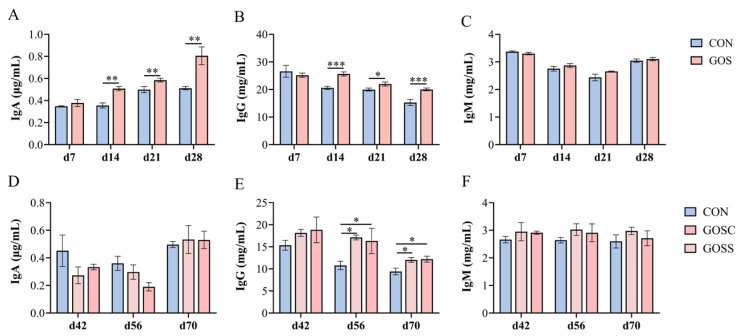
Early-life galacto-oligosaccharide (GOS) supplementation elevates serum immunoglobulin (Ig) levels in Holstein dairy calves. (**A**–**C**) Serum IgA, IgG, and IgM levels on days 7, 14, 21, and 28. (**D**–**F**) Serum IgA, IgG, and IgM levels on days 42, 56, and 70. Values are presented as Mean ± SEM. Different letters indicate significant differences between groups at the same time point. Asterisks indicate statistical significance: * *p* < 0.05, ** *p* < 0.01, *** *p* < 0.001. CON: control group, calves fed a basal diet without GOS supplementation throughout the study; GOS: calves supplemented daily with 10 g GOS from birth to day 28; GOSS: calves previously supplemented with 10 g GOS from birth to day 28, with GOS supplementation withdrawn thereafter; GOSC: calves supplemented daily with 10 g GOS continuously from birth to day 70.

As shown in Figure 1, calves fed GOS exhibited significantly higher IgA (*p* < 0.01; Figure 1A) and IgG (*p* < 0.05; Figure 1B) levels on days 14, 21, and 28, while no difference was detected on day 7 (*p* > 0.05). Serum IgM levels remained unchanged throughout the supplementation period (*p* > 0.05; Figure 1C). Notably, calves fed GOS maintained significantly higher IgG concentrations than the CON group on days 56 and 70 (*p* < 0.01; Figure 1E), even six weeks after GOS withdrawal. In contrast, IgA and IgM concentrations at these later time points did not differ between groups (*p* > 0.05; Figure 1D,F).

### 3.2. Effects of Dietary GOS Supplementation on Fecal Microbiome Composition on Day 28

**Figure 2:**
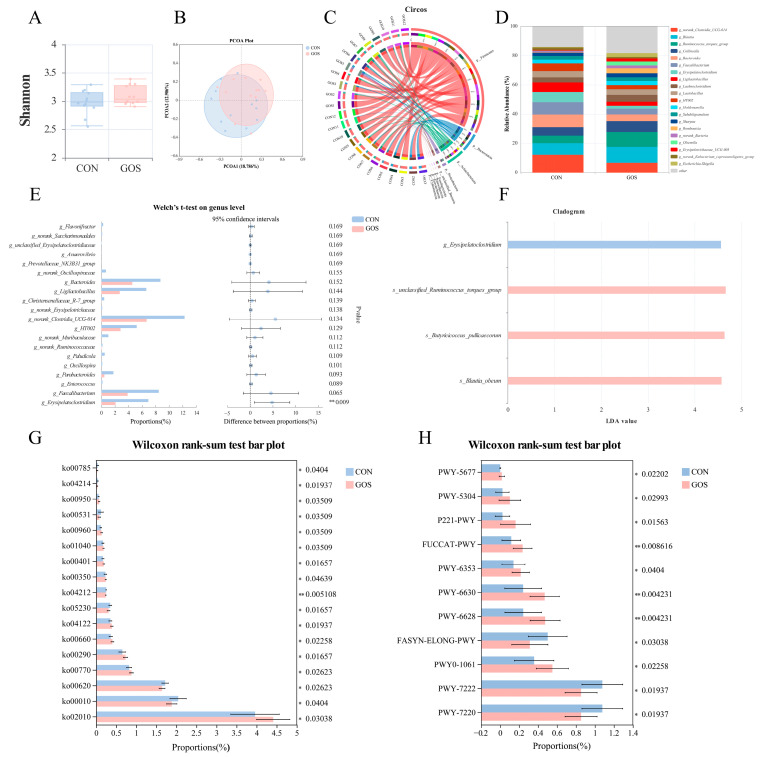
Galacto-oligosaccharide (GOS) supplementation modulates fecal microbial diversity and composition on day 28. (**A**) Shannon diversity index. (**B**) Principal coordinates analysis (PCoA) based on Bray–Curtis distance. (**C**,**D**) Taxonomic composition of the fecal microbiome at the phylum (**C**), and genus (**D**) levels. (**E**) Welch’s *t*-test identifying differential microbial taxa between the CON and GOS groups at the genus levels and (**F**) linear discriminant analysis effect size (LEfSe) identifying differential microbial taxa between the CON and GOS groups at the genus and species levels. Significantly different (**G**) KEGG level-3 pathways and (**H**) MetaCyc pathway between groups based on Wilcoxon rank-sum test. Asterisks indicate statistical significance: * *p* < 0.05, ** *p* < 0.01. CON: control group, calves fed a basal diet without GOS supplementation throughout the study; GOS: calves supplemented daily with 10 g GOS from birth to day 28.

On day 28, GOS supplementation did not notably affect fecal microbial α-diversity (*p* > 0.05; Figure 2A) or β-diversity (*p* > 0.05; Figure 2B, Table S2). However, taxonomic composition analysis revealed distinct shifts between groups. In the CON group, the dominant phyla included Firmicutes and Bacteroidota, whereas in the GOS group, Firmicutes and Actinobacteriota prevailed (Figure 2C). At the genus level, the CON group showed a higher relative abundance of *g_norank_Clostridia_UCG-014* (12.25%), *g_Bacteroides* (8.70%), and *g_Faecalibacterium* (8.45%), while the GOS group was enriched in *g_Blautia* (10.92%), *g_Ruminococcus_torques_group* (10.15%), and *g_Collinsella* (7.44%) (Figure 2D). Conversely, the relative abundance of *g_Erysipelatoclostridium* was markedly lower in GOS-fed calves (*p* = 0.009; Figure 2E), a result also supported by LEfSe analysis showing this taxon as enriched in CON animals (*p* < 0.05; Figure 2F). At the species level, *s_Blautia_obeum*, *s_Butyricicoccus_pullicaecorum*, and *s_unclassified_Ruminococcus_torques_group* species were significantly enriched in the GOS group (*p* < 0.05; Figure 2F).

Functional predictions using PICRUSt2, based on KEGG and MetaCyc databases, revealed differences in the inferred metabolic potential of the fecal microbiota between groups. Predicted KEGG pathways with higher relative abundances in the GOS group included tyrosine metabolism, pantothenate and CoA biosynthesis, unsaturated fatty acid biosynthesis, and branched-chain amino acid (valine, leucine, and isoleucine) biosynthesis, as well as several alkaloid biosynthesis pathways (e.g., tropane, pyridine, isoquinoline) (*p* < 0.05; Figure 2G). In contrast, the CON group showed higher activity in glycosaminoglycan degradation, glycolysis/gluconeogenesis, lipoic acid, and pyruvate metabolism (*p* < 0.05; Figure 2G). MetaCyc functional profiling further highlighted enrichment of amino acid synthesis pathways—including L-phenylalanine, L-tyrosine, and L-alanine biosynthesis—as well as fucose degradation and octane oxidation in GOS-fed calves (*p* < 0.05; Figure 2H). In contrast, the CON microbiota showed higher predicted activity in de novo nucleotide synthesis (adenosine and guanosine derivatives) and saturated fatty acid elongation (*p* < 0.05; Figure 2H). Overall, although GOS did not markedly change microbial diversity on day 28, it was associated with shifts in microbial taxonomic composition and predicted metabolic functions.

### 3.3. Post-Supplementation Effects of GOS Supplementation on the Fecal Microbiome Composition on Day 70

**Figure 3:**
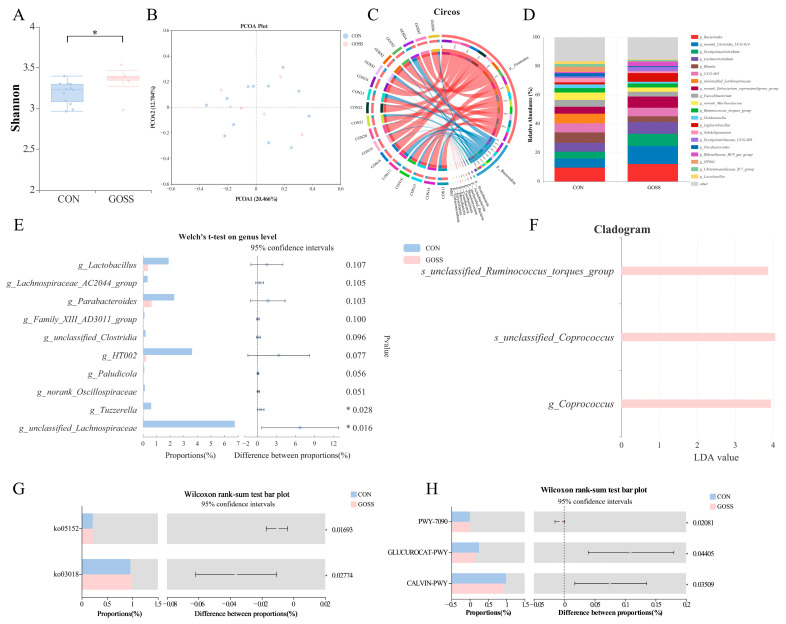
Persistent effects of early-life galacto-oligosaccharide (GOS) supplementation on fecal microbiota on day 70. (**A**) Shannon diversity index. (**B**) Principal coordinates analysis (PCoA) based on Bray–Curtis distance. (**C**,**D**) Taxonomic composition of the fecal microbiome at the phylum (**C**) and genus (**D**) levels. (**E**) Welch’s *t*-test identifying differential microbial taxa between the CON and GOS groups at the genus levels and (**F**) linear discriminant analysis effect size (LEfSe) identifying differential microbial taxa between the CON and GOS groups at the genus and species levels. Significantly different (**G**) KEGG level-3 pathways and (**H**) MetaCyc pathway between groups based on Wilcoxon rank-sum test. Asterisks indicate statistical significance: * *p* < 0.05. CON: control group, calves fed a basal diet without GOS supplementation throughout the study; GOSS: calves previously supplemented with 10 g GOS from birth to day 28, with GOS supplementation withdrawn thereafter.

Although GOS supplementation ceased on day 28, differences in the fecal microbiome were still detectable on day 70 between the GOSS group and CON calves. The Shannon diversity index was higher in the GOSS group than in the CON group (*p* < 0.05; Figure 3A), whereas no significant differences were observed in β-diversity based on PCoA and ANOSIM analyses (*p* > 0.05; Figure 3B, Table S2). At the phylum level, Firmicutes and Bacteroidota remained dominant in both groups (Figure 3C). At the genus level, *g_Bacteroides* (9.59%), *g_Blautia* (7.18%), and *g_unclassified_Lachnospiraceae* (6.75%) were the most abundant genera in the CON calves, whereas the calves in the GOSS group were dominated by *g_norank_Clostridia_UCG-014* (12.58%), *g_Bacteroides* (12.12%), and *g_Erysipelatoclostridium* (8.29%) (Figure 3D). Taxonomic comparison showed lower proportions of *g_Tuzzerella* and *g_unclassified_Lachnospiraceae* (*p* < 0.05; Figure 3E) in the GOSS group, whereas *g_Coprococcus*, *s_unclassified_Coprococcus*, and *s_unclassified_Ruminococcus_torques_group* were relatively enriched (*p* < 0.05; Figure 3F).

Functional prediction revealed that the RNA degradation pathway (ko03018) was upregulated in the GOSS group (*p* < 0.05; Figure 3G). According to MetaCyc analysis, the UDP-2,3-diacetamido-2,3-dideoxy-α-D-mannuronate biosynthesis route (PWY-7090) was also enhanced in the GOSS group, whereas the β-D-glucuronide and D-glucuronate degradation (GLUCUROCAT-PWY) and Calvin–Benson–Bassham cycle (CALVIN-PWY) were more active in control calves (*p* < 0.05; Figure 3H). Together, these findings suggest that even short-term GOS supplementation during early life can exert post-supplementation effects on microbial composition and functional potential.

### 3.4. Post-Supplementation Effects of GOS Supplementation on the Fecal Metabolic Profile on Day 70

**Figure 4:**
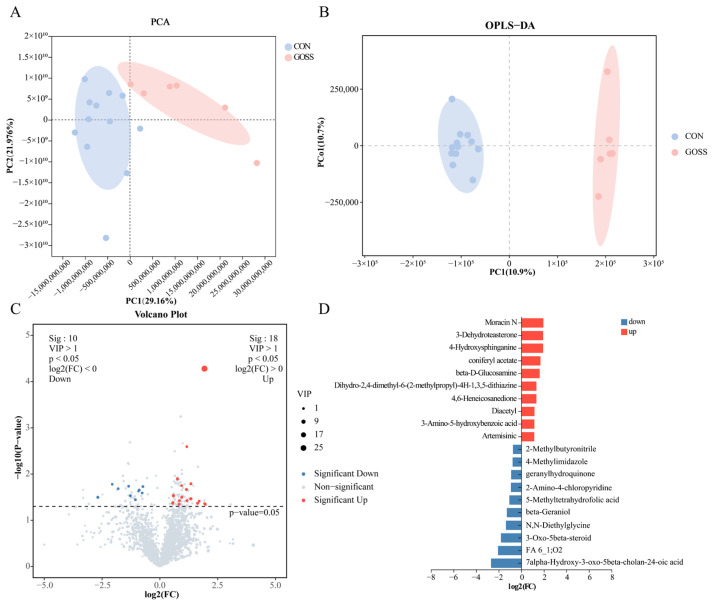
Persistent effects of early-life galacto-oligosaccharide (GOS) supplementation on fecal metabolomic profiles on day 70. (**A**) Principal component analysis (PCA). (**B**) Orthogonal partial least squares-discriminant analysis (OPLS-DA). (**C**) Volcano plot showing significantly different metabolites between the CON and GOSS groups based on *t*-tests (*p* < 0.05) and VIP scores (VIP > 1). (**D**) Bar plot of significant metabolites. CON: control group, calves fed a basal diet without GOS supplementation throughout the study; GOSS: calves previously supplemented with 10 g GOS from birth to day 28, with GOS supplementation withdrawn thereafter.

**Figure 5:**
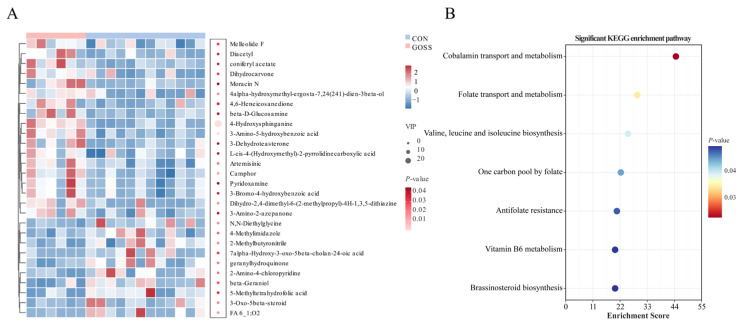
Differential fecal metabolites and enriched KEGG pathways after galacto-oligosaccharide (GOS) supplementation at day 70. (**A**) Combined heatmap of significant metabolites and variable importance in projection (VIP) bubble plot. (**B**) Bubble plot of significantly enriched KEGG pathways for differential metabolites. Significance was defined by a *p*-value < 0.05. CON: control group, calves fed a basal diet without GOS supplementation throughout the study; GOSS: calves previously supplemented with 10 g GOS from birth to day 28, with GOS supplementation withdrawn thereafter.

Untargeted fecal metabolomics identified 2740 metabolites in the CON and GOSS groups. The PCA and OPLS-DA revealed clear group separation (Figure 4A,B) with high model reliability (R^2^Y = 0.987 and Q^2^ = 0.597; Figure S1A). Multivariate analysis combining VIP scores and *t*-tests identified 28 metabolites differing between groups (*p* < 0.05, VIP > 1; Figure 4C). Eighteen metabolites were elevated, while ten were reduced in the GOSS group. Among the increased compounds were sphingolipid intermediates (e.g., 4-Hydroxysphinganine), pyridoxamine, and phenolic derivatives, suggesting shifts in lipid and vitamin metabolism (*p* < 0.05, Figure 4D). Downregulated metabolites included 7alpha-Hydroxy-3-oxo-5beta-cholan-24-oic acid, beta-Geraniol, 5-Methyltetrahydrofolic acid, geranylhydroquinone, 2-Methylbutyronitrile, N,N-Diethylglycine, 3-Oxo-5beta-steroid, 2-Amino-4-chloropyridine, 4-Methylimidazole, and FA 6_1;O2 (*p* < 0.05, Figure 4D). A heatmap visualization confirmed the distinct metabolomic signatures between the CON and GOSS groups (*p* < 0.05, VIP > 1; Figure 5A). Pathway enrichment of these differential metabolites pointed to significant involvement in vitamin-related and amino acid metabolic pathways, including cobalamin and folate metabolism, branched-chain amino acid biosynthesis, one-carbon metabolism, vitamin B6 metabolism, and brassinosteroid biosynthesis (*p* < 0.05; Figure 5B). These results demonstrate that early GOS supplementation produces post-supplementation change in the fecal metabolic profile, particularly in vitamin- and amino acid-related pathways.

### 3.5. Post-Supplementation Effects of GOS Supplementation on the Serum Metabolic Profile on Day 70

**Figure 6:**
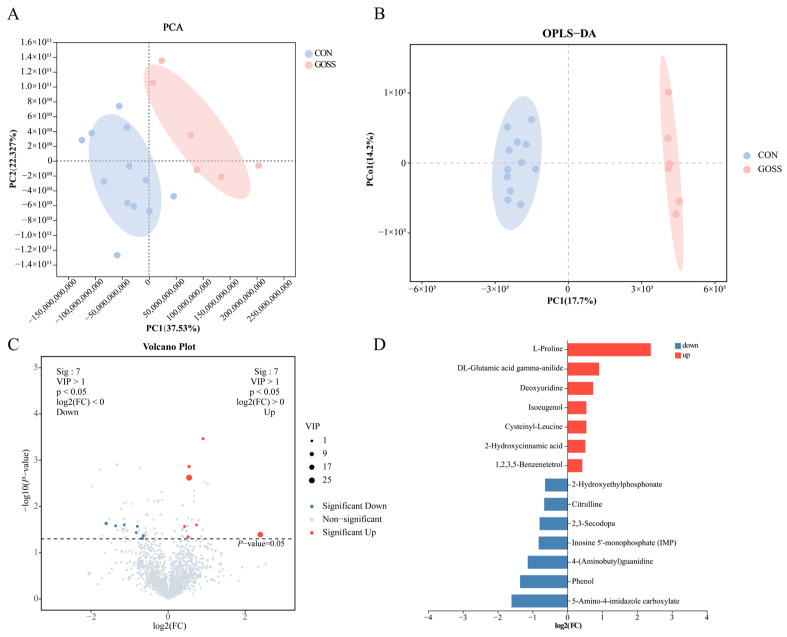
Persistent effects of early-life galacto-oligosaccharide (GOS) supplementation on serum metabolomic profiles on day 70. (**A**) Principal component analysis (PCA). (**B**) Orthogonal partial least squares-discriminant analysis (OPLS-DA). (**C**) Volcano plot showing significantly different metabolites between the CON and GOSS groups based on *t*-tests (*p* < 0.05) and VIP scores (VIP > 1). (**D**) Bar plot of significant metabolites. CON: control group, calves fed a basal diet without GOS supplementation throughout the study; GOSS: calves previously supplemented with 10 g GOS from birth to day 28, with GOS supplementation withdrawn thereafter.

**Figure 7:**
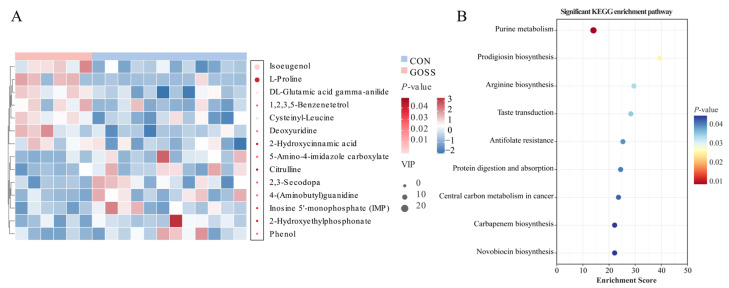
Differential serum metabolites and enriched KEGG pathways after galacto-oligosaccharide (GOS) supplementation on day 70. (**A**) Combined heatmap of significant metabolites and variable importance in projection (VIP) bubble plot. (**B**) Bubble plot of significantly enriched KEGG pathways for differential metabolites. Significance was defined by a *p*-value < 0.05. CON: control group, calves fed a basal diet without GOS supplementation throughout the study; GOSS: calves previously supplemented with 10 g GOS from birth to day 28, with GOS supplementation withdrawn thereafter.

Serum metabolomics detected 1870 metabolites across the two groups. The PCA and OPLS-DA revealed clear separation, with high model performance (R^2^Y = 0.987; Q^2^ = 0.778; Figure S1B). Statistical analysis using *t*-tests and VIP scores identified 14 significantly different metabolites between groups (*p* < 0.05, VIP > 1; Figure 6C). Among these, seven metabolites were upregulated and seven were downregulated in the GOSS group compared to the CON group. Specifically, the upregulated metabolites in the GOSS group were mainly amino acid derivatives, phenolic antioxidants, and nucleoside intermediates, while downregulated metabolites were primarily purine- and arginine-related compounds and small organic acids (*p* < 0.05; Figure 6D). The heatmap visualization further highlighted the distinct serum metabolic signatures between groups (*p* < 0.05, VIP > 1; Figure 7A). Pathway enrichment analysis indicated that these metabolic alterations were concentrated in nine pathways: purine metabolism, prodigiosin biosynthesis, arginine biosynthesis, taste transduction, antifolate resistance, protein digestion and absorption, carbapenem biosynthesis, and novobiocin biosynthesis (*p* < 0.05; Figure 7B). Collectively, these findings suggest that early GOS supplementation is associated with post-supplementation change in the serum metabolic profile, with potential implications for host nutrient utilization and immune function.

### 3.6. Relationships Between Fecal Microbial, Fecal/Serum Metabolites, and Immunoglobulin Levels

**Figure 8:**
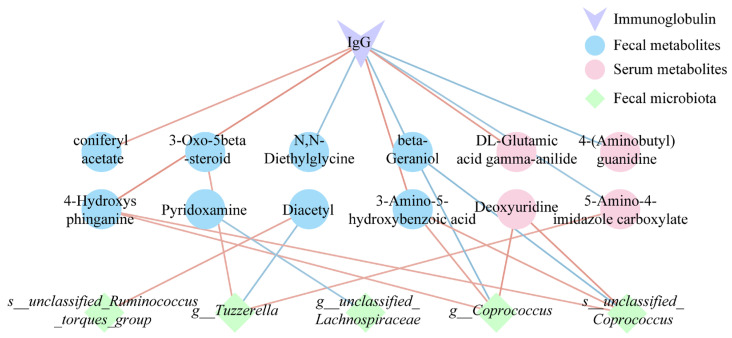
Network diagram illustrating the associations between fecal microbial, fecal/serum metabolites, and immunoglobulin levels (|R| > 0.50, *p* < 0.05). The color of lines denotes direction of Spearman’s correlation (red: R > 0; blue: R < 0; color depth indicates the value of the coefficient). IgG: immunoglobulin G.

To explore potential links among gut microbiota, metabolic profiles, and immune responses, Spearman’s correlation analyses were conducted between differential fecal microbial taxa and fecal/serum metabolites, as well as between differential metabolites and serum immunoglobulin levels. The results are summarized in correlation heatmaps (Figure S2). As shown in Figure S2A, the relative abundance of *g_Tuzzerella* exhibited a negative correlation with Diacetyl and a positive correlation with 3-Oxo-5beta-steroid (|R| > 0.50, *p* < 0.05). The relative abundance of *g_unclassified_Lachnospiraceae* was negatively associated with Pyridoxamine (|R| > 0.50, *p* < 0.05), whereas that of *s_unclassified_Ruminococcus_torques_group* showed a positive correlation with Diacetyl (|R| > 0.50, *p* < 0.05). Notably, both *g_Coprococcus* and *s_unclassified_Coprococcus* were positively correlated with 4-Hydroxysphinganine and 3-Amino-5-hydroxybenzoic acid (|R| > 0.50, *p* < 0.05), while exhibiting negative correlations with beta-Geraniol (|R| > 0.50, *p* < 0.01). Further analysis revealed that metabolites positively associated with *Coprococcus*, including 4-Hydroxysphinganine and 3-Amino-5-hydroxybenzoic acid, were also positively correlated with serum IgG level (|R| > 0.50, *p* < 0.01; Figure S2B). As shown in Figure S2C,D, the relative abundance of *g_Tuzzerella* was positively correlated with 5-Amino-4-imidazole carboxylate (|R| > 0.50, *p* < 0.05), a metabolite that was in turn negatively associated with serum IgG level (|R| > 0.50, *p* < 0.05). In addition, both *s_unclassified_Coprococcus* and *g_Coprococcus* were positively correlated with Deoxyuridine (|R| > 0.50, *p* < 0.01). Regarding serum metabolites, serum IgG level was negatively associated with 4-(Aminobutyl)guanidine (|R| > 0.50, *p* < 0.05) and positively associated with DL-Glutamic acid gamma-anilide (|R| > 0.50, *p* < 0.01). Collectively, these findings suggest that the changes in specific microbial taxa induced by early GOS supplementation are associated with alterations in metabolite signatures and IgG levels (Figure 8).

## 4. Discussion

Although the health-promoting effects of GOS are well-documented, the underlying regulatory mechanisms between microbial modulation and host immunity have yet to be fully elucidated. Our previous studies have demonstrated that GOS supplementation reduced diarrhea incidence, improved the average daily gain, and enhanced feed efficiency during early life. In addition, GOS supplementation improved rumen microbiome composition and immune function in Holstein calves, with benefits extending beyond the supplementation period [13,14]. In this context, this study examined the post-supplementation effects of early GOS supplementation on circulating immunoglobulin levels, intestinal microbial ecology, and metabolic profiles in Holstein calves.

GOS supplementation elevated serum IgA and IgG concentrations during early development, with IgG remaining high even six weeks after supplementation ended. The persistent elevation in IgG, a crucial component of systemic immunity, is consistent with findings from Yu et al. [14], who also observed immune enhancement via increased immunoglobulin levels. The mechanisms behind these effects likely involve the role of GOS in enhancing gut barrier integrity [34] and modulating gut microbial colonization [7], where much of the immune system develops. The gastrointestinal microbiota acts as a vital organ that shapes immune function, metabolism, and intestinal homeostasis [35].

GOS supplementation induced distinct shifts in gut microbiota composition, both during and after the intervention. On day 28, GOS supplementation did not significantly alter alpha diversity but induced notable changes in microbial composition. Calves receiving GOS exhibited a higher abundance of health-associated genera such as *Blautia, Butyricicoccus_pullicaecorum,* and *Ruminococcus*, while potentially harmful *Erysipelatoclostridium* declined. These shifts are consistent with previous studies showing that GOS selectively promotes saccharolytic- and butyrate-producing bacteria that support mucosal health and immune responses [9,36]. The increased abundances of *Blautia, Butyricicoccus_pullicaecorum*, and *Ruminococcus* are likely associated with enhanced gut barrier integrity and immune modulation. *Blautia*, an anaerobic bacterium, promotes fiber digestion and SCFA production, maintaining intestinal homeostasis [37]. *Butyricicoccus pullicaecorum*, a butyrate-producing bacterium, is known to improve feed conversion and reduce pathogenic bacteria, highlighting its potential as a probiotic [38]. Similarly, *Ruminococcus* species, which degrade mucin, play a key role in gut health through SCFA production [39].

Even six weeks after GOS supplementation ceased, differences in microbial composition were still detectable on day 70. The increased Shannon index in the GOSS group indicated improved microbial richness and diversity, while LEfSe analysis identified enrichment of *g_Coprococcus*, *s_unclassified_Coprococcus*, and *s_unclassified_Ruminococcus_torques_group*, both of which are linked to SCFA production and gut homeostasis [40,41]. These post-supplementation effects of fecal microbiome indicate that early nutritional intervention with GOS can establish a favorable intestinal ecosystem that promotes gastrointestinal stability—consistent with previous observations of enhanced growth and reduced diarrhea [13]. GOS supplementation also induced significant changes in fecal microbial metabolic functions. On day 28, GOS enhanced pathways related to amino acid biosynthesis, unsaturated fatty acid production, and coenzyme metabolism, suggesting a shift toward improved nutrient utilization and microbial activity. By day 70, despite the cessation of supplementation, similar pathways remained active, indicating that the microbiota continued to influence host metabolism. In addition, the correlation analysis indicates coordinated associations among specific microbial taxa, metabolites, and serum IgG levels, supporting a potential microbiota–metabolite–immune interaction that underlies the post-supplementation immunomodulatory effects of early-life GOS supplementation. Collectively, the results highlight that GOS remodels microbial composition and functionality, which is linked to metabolic balance, intestinal integrity, and immune responses in dairy calves.

In addition to microbial composition, GOS supplementation induced persistent shifts in both fecal and serum metabolic profiles. The fecal metabolome showed upregulation of metabolites such as pyridoxamine, 4-hydroxysphinganine, and branched-chain amino acid (BCAA)-related intermediates. KEGG enrichment revealed pathways related to vitamin B6, cobalamin, folate metabolism, and BCAA biosynthesis, which are all crucial for immune function and metabolic health. These results suggest that GOS supplementation alters microbial metabolic capacity, promoting the production of essential vitamins and amino acids that support host health [42]. Disruption of microbial vitamin B biosynthesis has been associated with impaired microbial and host metabolism [43,44]. Indeed, ongoing studies in our laboratory have also found functional enrichment of functional modules related to B vitamin biosynthesis in the rumen of calves fed with GOS through metagenomics, including biotin biosynthesis (M00573, M00950), tetrahydrofolate biosynthesis (M00840), and pantothenic acid biosynthesis (M00913) (unpublished data). Studies have shown that BCAA are central to regulating energy turnover, nutrient metabolism, and immune homeostasis [45]. Wang et al. [46] reported that the probiotic strain AR-9 modulates amino acid metabolism, increases gut microbial diversity and abundance, and optimizes microbial community structure, thereby contributing to host health. Similar metabolic regulation has been observed in other prebiotic models; for instance, Schisandra chinensis bee pollen adjusts microbial composition and modulates carbohydrate and amino acid metabolism [47]. In the present study, GOS supplementation appeared to enhance the microbial capacity for vitamin cofactor metabolism (vitamin B6, folate, cobalamin) and amino acid biosynthesis. Furthermore, the observed reduction in 5-methyltetrahydrofolic acid levels, together with the enrichment of “antifolate resistance” pathways, points to alterations in one-carbon metabolism and folate turnover—key processes linked to DNA synthesis, cell proliferation, and immune regulation [48,49]. Serum metabolomics further revealed elevated amino acids (proline, cysteinyl-leucine) and bioactive phenolics (isoeugenol, 2-hydroxycinnamic acid), alongside decreased inosine monophosphate, indicating changes in protein synthesis, antioxidant capacity, and purine metabolism. Bioactive phenolics like isoeugenol and 2-hydroxycinnamic acid are known for their antioxidant and antimicrobial activities [50,51], which may enhance immune function and metabolic regulation.

In summary, early life represents a critical window for immune maturation and gut microbial establishment in dairy calves, during which the immune system, microbial communities, and host metabolism undergo rapid development. The present findings suggest that early-life GOS supplementation may influence this process by selectively promoting microbial taxa with metabolic capacities related to amino acid biosynthesis, vitamin cofactor metabolism, and nitrogen utilization. These microbiota-derived metabolites may support immunoglobulin production and immune-related functions during this sensitive developmental stage. Consistent with previous evidence showing that early-life probiotic or prebiotic interventions can modulate gut microbial communities and immune development [52], GOS supplementation in the current study was associated with shifts in microbial composition and metabolic pathways, as well as circulating immunoglobulin levels. By promoting beneficial gut microbiota and SCFA production, GOS may influence immune cell maturation and activity, including B cells that are responsible for immunoglobulin production [53,54]. Given the high plasticity of the calf gut ecosystem before weaning, such early microbial and metabolic modulation may exert effects that remain detectable beyond the supplementation period.

In a previous study using the same cohort of calves, early-life GOS supplementation improved average daily gain and feed efficiency, and also reduced diarrhea incidence [13,14]. The current results provide mechanistic context for these production outcomes by linking early GOS exposure to immune-related indicators and metabolic pathways associated with nutrient utilization. Notably, the sustained elevation in IgA and IgG suggests enhanced immune function, which may contribute to reduced disease susceptibility and improved resilience during the high-risk preweaning period. Concurrent enrichment of pathways related to vitamin cofactors, amino acid biosynthesis, and purine and arginine metabolism further suggests microbial–host metabolic adaptation that may support growth and feed utilization. Although effects persisted beyond the supplementation period, the experimental duration was limited to the preweaning stage, and longer-term follow-up studies are required to determine whether these early-life changes extend into later production phases. Taken together with previous findings, GOS supplementation of 10 g/day from birth to day 28 appears to be a feasible early-life nutritional strategy for dairy calves. However, optimization of dosage, duration, and long-term outcomes warrants further investigation. From a practical perspective, early-life GOS supplementation may contribute to improved developmental stability and reduced disease susceptibility during the preweaning period by supporting immune-related and metabolic processes.

## 5. Conclusions

Early-life dietary supplementation with GOS in Holstein calves was associated with alterations in immune-related indicators, as reflected by increased serum IgA and IgG levels during the supplementation period, with sustained IgG elevation after supplementation ceased. Early GOS supplementation was also associated with shifts in intestinal microbial composition and predicted metabolic functions, accompanied by differences in fecal and serum metabolomic profiles detected after supplementation ceased. These changes were mainly linked to pathways involving vitamin cofactors, amino acid turnover, and purine–arginine metabolism. Together, these findings indicate that GOS provides post-supplementation effects for immunoglobulin levels and metabolite profiles beyond the intervention period, supporting its potential as an early-life nutritional strategy to improve calf health and productivity, and to reduce disease susceptibility through enhanced immune preparedness. Future studies should elucidate the mechanistic connections between microbial and metabolic changes and immune outcomes, determine the persistence of these effects into adulthood, and evaluate their impacts on lifetime productivity and health. The integration of multi-omics analyses with targeted mechanistic experiments will be critical to fully realize the potential of GOS in livestock production systems.

## Data Availability

The raw 16S rRNA sequencing data have been deposited in the NCBI Sequence Read Archive (SRA) under accession number PRJNA751971 (https://www.ncbi.nlm.nih.gov/bioproject/PRJNA751971, accessed on 8 May 2025).

## Supplementary Materials

The following supporting information can be downloaded at https://www.mdpi.com/article/10.3390/ani16010126/s1; Table S1. The nutrient compositions of the starter and milk (%). Table S2. Difference in the beta diversity index of microbiome composition on day 28 and 70. Figure S1: OPLS-DA model validation plot of fecal and serum metabolomic profiles; Figure S2: Correlations between fecal microbial, fecal/serum metabolites, and immunoglobulin levels.

## Abbreviations

The following abbreviations are used in this manuscript:

GOS	Galacto-oligosaccharides
IgA	Immunoglobulin A
IgG	Immunoglobulin G
IgM	Immunoglobulin M
SCFAs	short-chain fatty acids
ASVs	Amplicon sequence variants
LDA	Linear discriminant analysis
LefSe	Linear discriminant analysis effect size
QC	Quality control
PCA	Principal component analysis
OPLS-DA	Orthogonal partial least squares-discriminant analysis
VIP	Variable importance in projection
