# Supplementary material for: Early-Life Galacto-Oligosaccharide Supplementation Induces Persistent Immunoglobulin and Metabolic Alterations in Holstein Dairy Calves by Shaping Gut Microbiota
Source: Animals (Basel). 2026 Jan 1;16(1):126. doi: 10.3390/ani16010126 (PMC12785093; doi:10.3390/ani16010126)
Supplement: Supplementary file 1 [file animals-16-00126-s001.zip › Final Supplementary material/Supplementary material.pdf]

Type of the Paper (Article) ***Supplementary material***

Early-Life Galacto-Oligosaccharide Supplementation Induces Persistent Immunoglobulin and Metabolic Alterations in Holstein Dairy Calves by Shaping Gut Microbiota

Qi Huang, Meinan Chang and Peng Sun *

State Key Laboratory of Animal Nutrition and Feeding, Institute of Animal Science, Chinese Academy of Agricultural Sciences, Beijing 100193, China; workhq@163.com (Q.H.); meinan0616@126.com (M.C.)

* Correspondence: sunpeng02@caas.cn

Table S1. The nutrient compositions of the starter and milk (%).

Item	Milk, as-is basis	Item	Starter, dry matter basis
Density, kg/L	1.03	DM, %	90.1
Milk protein	3.13	CP, %DM	18.8
Milk fat	3.87	EE, %DM	5.55
Lactose	5.09	NDF, %DM	7.95
TS	12.2	ADF, %DM	3.09
SNF	9.54	Ash, %DM	8.60

Abbreviations: TS = total solids; SNF = solids of non-fat; DM = dry matter; CP = crude protein; EE = ether extract; NDF = neutral detergent fiber; ADF = acid detergent fiber.

Table S2. Difference in the beta diversity index of microbiome composition on day 28 and 70.

Method	Statistic	*P*-value
D28		
ANOSIM	R（0.0250）	0.275
D70		
ANOSIM	R（-0.1495）	0.95

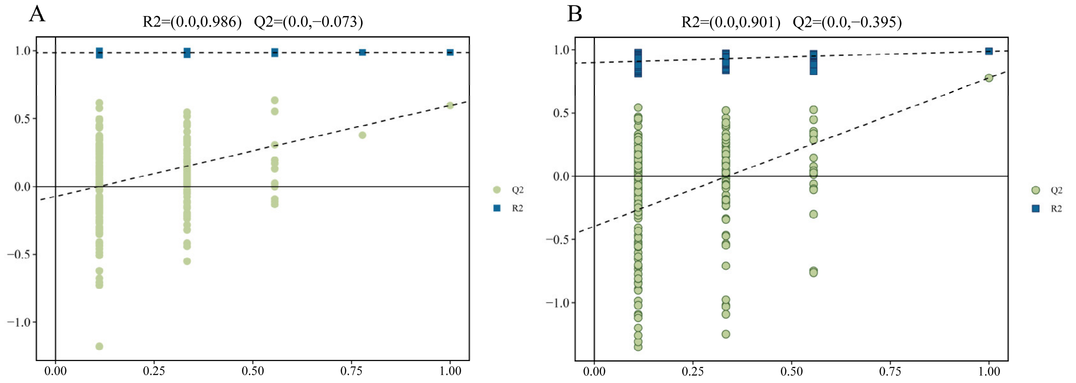

Supplementary Fig. S1. OPLS-DA model validation plot of fecal (A) and serum (B) metabolomic profiles. The x-axis represents the permutation retention ratio (i.e., the proportion of Y variable order retained), with 1 indicating the original model. The y-axis shows the R^2 (blue square) and Q^2 (green circle) values from each permutation. Dotted lines represent regression lines for R^2 and Q^2.

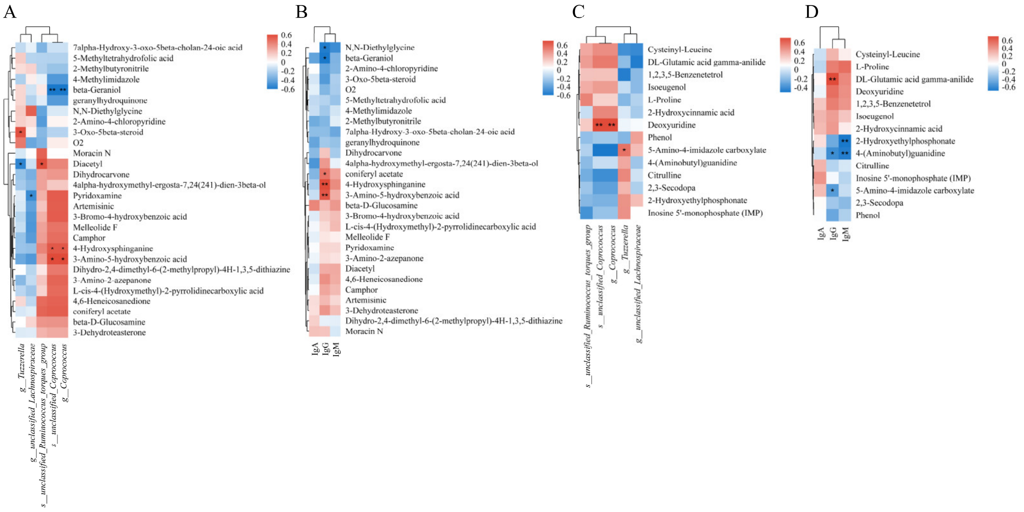

Supplementary Fig. S2. Correlations between fecal microbial, fecal/serum metabolites, and immunoglobulin levels. Heatmaps showing Spearman's rank correlation coefficients between (A) differential fecal microbial taxa and fecal metabolites, (B) differential fecal metabolites and serum immunoglobulin levels, (C) differential fecal microbial taxa and serum metabolites, and (D) differential serum metabolites and serum immunoglobulin levels. Color intensity represents the strength and direction of the correlation (red: positive; blue: negative). Asterisks indicate statistical significance: * $P < 0.05$, ** $P < 0.01$. IgA: Immunoglobulin A; IgG: Immunoglobulin G; IgM: Immunoglobulin M.
