# Supplementary figures and images for: Early-Life Galacto-Oligosaccharide Supplementation Induces Persistent Immunoglobulin and Metabolic Alterations in Holstein Dairy Calves by Shaping Gut Microbiota
Source: Animals (Basel). 2026 Jan 1;16(1):126. doi: 10.3390/ani16010126 (PMC12785093; doi:10.3390/ani16010126)

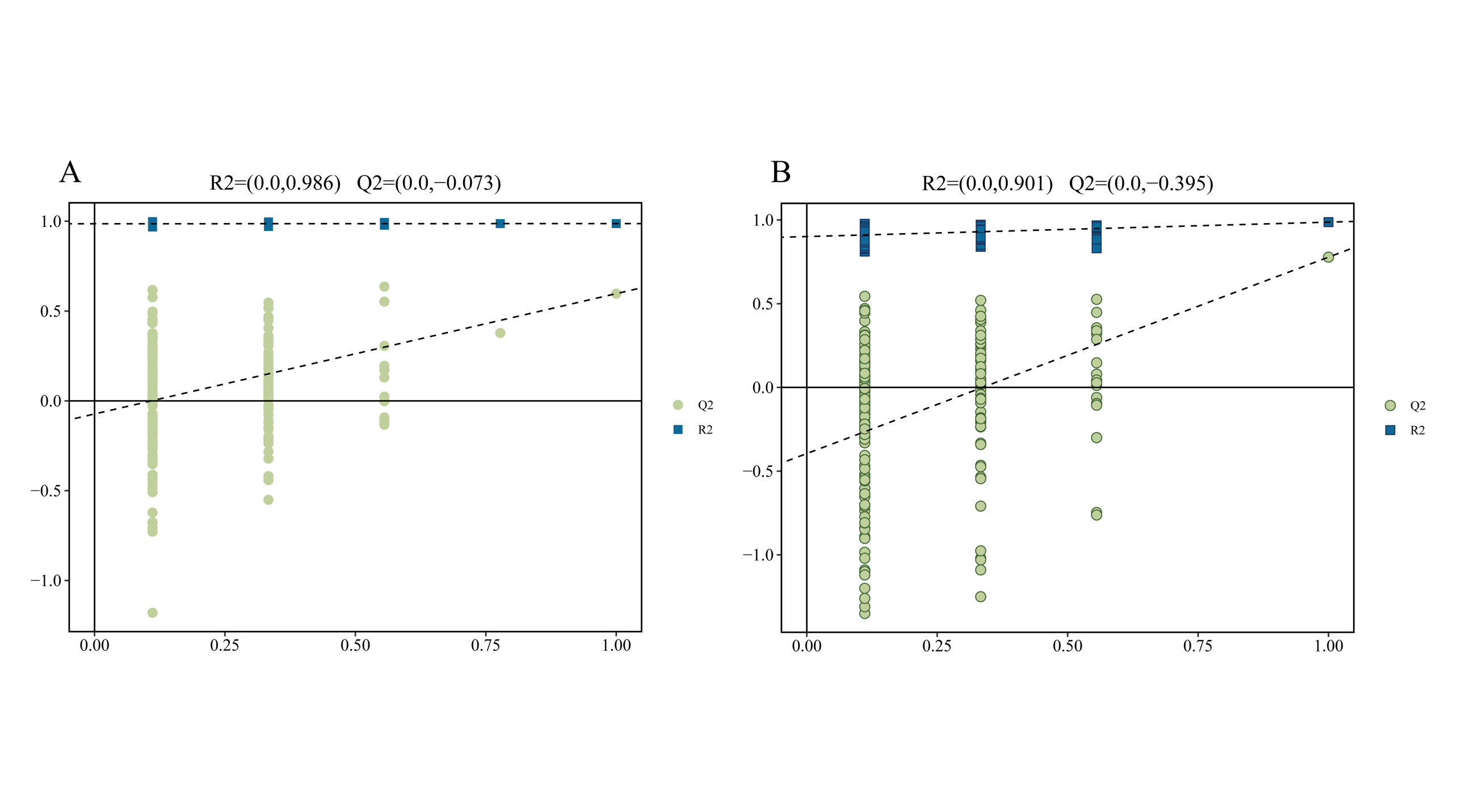

Supplement: Supplementary file 1 [file animals-16-00126-s001.zip › Final Supplementary material/Fig S1.tif]

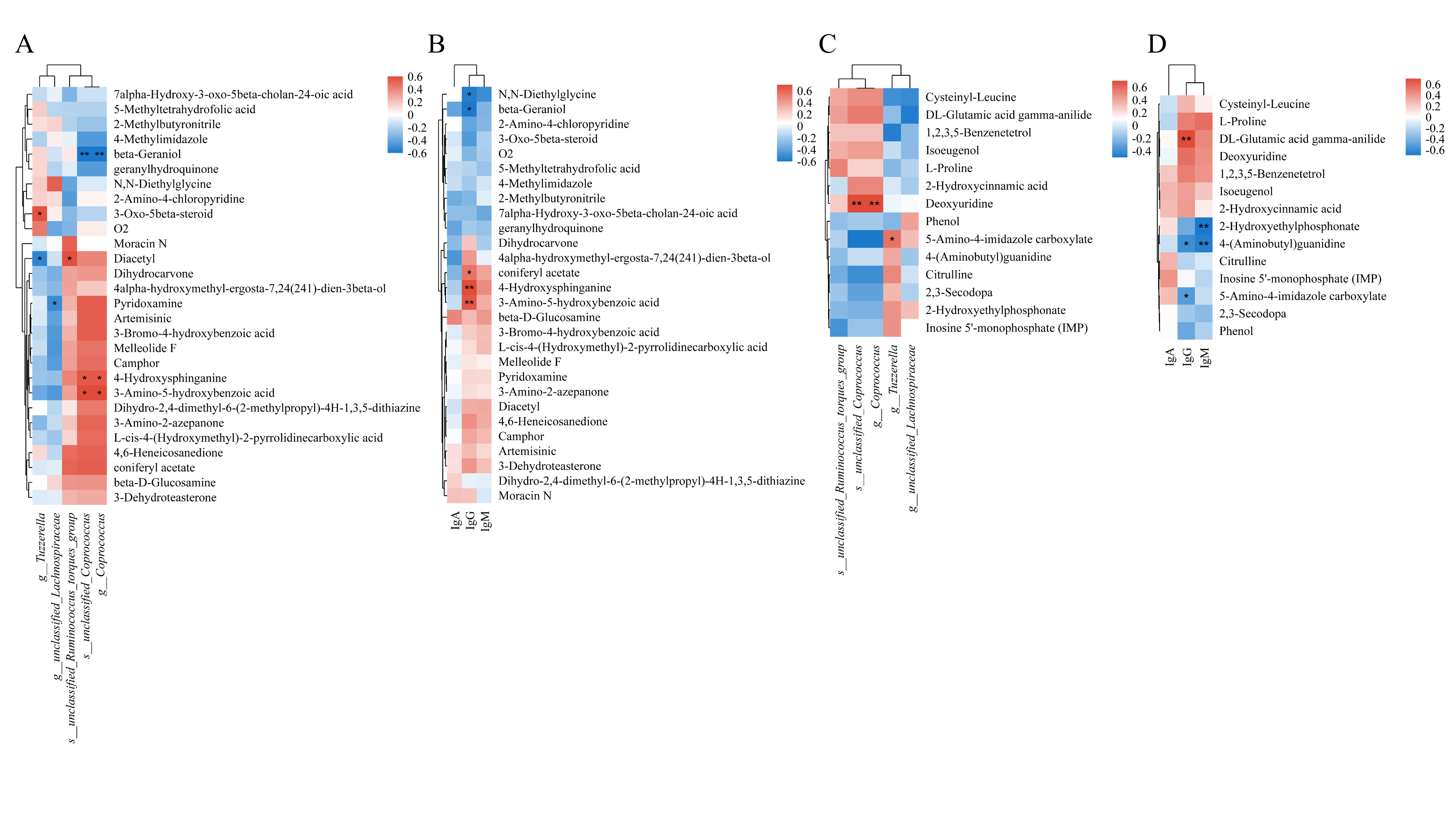

Supplement: Supplementary file 1 [file animals-16-00126-s001.zip › Final Supplementary material/Fig S2.tif]
